# *Capillaria* Ova and Diagnosis of *Trichuris trichiura* Infection in Humans by Kato-Katz Smear, Liberia

**DOI:** 10.3201/eid2408.180184

**Published:** 2018-08

**Authors:** Kerstin Fischer, Abakar Gankpala, Lincoln Gankpala, Fatorma K. Bolay, Kurt C. Curtis, Gary J. Weil, Peter U. Fischer

**Affiliations:** Washington University School of Medicine, St. Louis, Missouri, USA (K. Fischer, K.C. Curtis, G.J. Weil, P.U. Fischer);; National Public Health Institute of Liberia, Charlesville, Liberia (A. Gankpala, L. Gankpala, F.K. Bolay)

**Keywords:** soil-transmitted helminths, diagnosis, *Capillaria*, qPCR, *Trichuris trichiura*, parasites

## Abstract

We examined human stool samples from Liberia for soil-transmitted helminth ova by Kato-Katz smear and by quantitative PCR. Twenty-five samples were positive for *Trichuris trichiura* by smear but negative by quantitative PCR. Reexamination of samples showed that they contained *Capillaria* eggs that resemble *T. trichiura* in Kato-Katz smears.

Kato-Katz smears are the most commonly used diagnostic tool for detecting and quantifying soil-transmitted helminth (STH) infections in field surveys (*1*). Although this method has some shortcomings, its advantages are field suitability and fast microscopic enumeration of worm eggs. Whereas sensitivity is low for light infections because of the small amount of stool examined (≈41 mg), the specificity of Kato-Katz for diagnosis of *Ascaris lumbricoides* and *Trichuris trichiura* infection is considered to be high (*2*). In contrast, hookworm eggs are difficult to differentiate by morphology, but quantitative PCR (qPCR) enables differentiation among *Necator americanus*, *Ancylostoma duodenale,* and *A. ceylanicum* eggs (*3*–*5*).

Among helminth eggs found in human feces, the barrel-shaped eggs of *T. trichiura* worms are considered to be characteristic, with a length of 50–55 µm, a width of 22–24 µm, and clearly protruding bipolar plugs (*6*). Similar eggs of other members of the *Trichiuridae* family may be differentiated from *T. trichiura* eggs by size and morphology when observed at high magnification, but these eggs have rarely been found in human fecal samples (*7*–*9*). Therefore, the presence of eggs of zoonotic members of the *Trichiuridae* family is generally not considered a confounder for detecting *T. trichiura* by Kato-Katz smear.

## The Study

To assess the effect of mass drug administration using ivermectin and albendazole for the elimination of lymphatic filariasis on STH prevalence and intensity, we collected stool samples over a period of 3 years in 2 different areas in Foya district (Lofa County) in northwestern Liberia and in Harper district (Maryland County) in southeastern Liberia (*10*). We examined a single stool sample per subject by microscopy (magnification ×100) with duplicate Kato-Katz smears (41 mg template). We preserved aliquots of randomly selected specimens on FTA cards (GE Healthcare, Little Chalfont, UK) or in RNAlater (ThermoFisher, Waltham, MA, USA) and shipped them to Washington University School of Medicine (St. Louis, MO, USA) for analysis by qPCR. Two experienced microscopists (L.G., A.T. Momolu) examined the samples by Kato-Katz smear in both study areas. For detection of STH by qPCR, we extracted DNA from ≈100 mg of stool and tested it as described by Pilotte et al. (*5*) with a Quantstudio 6 Flex Thermocycler (Applied Biosystems, Carlsbad, CA, USA) and TaqMan Fast Advanced Mastermix (Applied Biosystems). We used the following primers and probes to detect *Schistosoma mansoni* DNA: forward primer 5′-TGTGGGAGTCTTTGGTTGTT-3′, reverse primer 5′-CAACATGACTGGGAACAGGA-3′, probe 5′-AGGTTCAGGTGG/ZEN/GTGTGTTACGAA-31ABkFQ-3′.

We tested 353 stool samples from Foya district by Kato-Katz smear; 31 (8.8%) were positive for *A. lumbricoides* eggs, 231 (65.4%) for hookworm eggs, 27 (7.6%) for *T. trichiura*–like eggs, and 276 (78.2%) for *S. mansoni* eggs. We tested 225 samples from Harper district by Kato-Katz smear; 163 (72.4%) were positive for *A. lumbricoides* eggs, 65 (28.9%) for hookworm eggs, and 51 (22.7%) for *T. trichiura* eggs (Table 1). There was good agreement between the results of the Kato-Katz and qPCR tests for the specimens from Harper (80.5%–91.6%), but generally qPCR had higher sensitivity. Our results were consistent with results previously reported with samples from other areas (*3*,*11*). Agreement between the 2 diagnostic tests for samples from Foya ranged from 77.3% to 92.9%, but the sensitivity of the qPCR was unexpectedly low, a finding that was especially true for *Ascaris* and *Trichuris* infection (Table 1). Whereas samples positive for *Ascaris* by Kato-Katz but negative by qPCR had low egg counts, samples positive for *Trichuris* by Kato-Katz but negative by qPCR had higher counts; 7 samples contained >1,000 barrel-shaped eggs/g of stool (Table 2). We repeated DNA extraction and qPCR and also used an alternative qPCR for *T. trichiura* (*3*), but these tests did not improve the agreement between microscopy and qPCR results.

**Table 1:** Comparison of sensitivity of Kato-Katz smear and quantitative PCR results for 778 stool samples tested for soil-transmitted helminths, Foya and Harper districts, Liberia

Site and species	No. positive*	Kato-Katz smear sensitivity, %	qPCR sensitivity, %	McNemar p value
Foya district, n = 353				
* Ascaris lumbricoides*	34	91.2	17.6	<0.0001
Hookworm†	247	93.5	83.4	<0.0001
* Trichuris trichiura*	27	100	7.4	<0.0001
* Schistosoma mansoni*	307	89.9	84.0	0.0573
Harper district, n = 225				
* A. lumbricoides*	180	90.6	98.9	0.0013
Hookworm†	99	65.7	89.9	0.0005
* T. trichiura*	86	59.3	94.2	0.0001

*Samples that tested positive by either method. †Hookworm was *Necator americanus.* No *Ancylostoma duodenale* was detected.

**Table 2:** Demographics and Kato-Katz and qPCR results for patients positive for *Trichuris trichiura* infection by microscopy, Liberia*

Demographics					Microscopy, epg					qPCR, cycle threshold			
Year	Patient no.	Age, y/sex	Village		Tt	Al	Hk	Sm		Tt	Al	Na	Sm
2014	P320529	45/F	Yallahun		576	0	360	24		Neg	Neg	31.7	30.5
	P320683	35/F	Kpombu		12	0	0	0		Neg	Neg	Neg	28.4
	P320695	16/M	Kpombu		24	0	0	72		Neg	Neg	Neg	23.8
	P320620	15/M	Foya-Dundu		12	120	0	288		Neg	Neg	32.2	23.5
	P320746	9/F	Bandenin		24	0	0	0		Neg	Neg	Neg	26.51
	P320452	7/F	Felaloe		12	0	0	120		Neg	Neg	Neg	23.9
	P320596	6/F	Foya-Dundu		12	0	0	90		Neg	Neg	Neg	27.6
	P320656	6/F	Kpombu		120	0	0	504		Neg	Neg	Neg	21.3
2016	P331772	36/M	Kpormbu		3,048	0	12	24		Neg	Neg	Neg	Neg
	P331921	35/M	Felaloe		60	0	0	12		Neg	Neg	Neg	Neg
	P331783	34/F	Kpormbu		420	0	0	0		Neg	Neg	Neg	Neg
	P330724	26/M	Keyabendu		4,224	0	0	456		Neg	Neg	Neg	30.4
	P331791	6/F	Kpormbu		12	0	156	12		Neg	Neg	33.1	33.4
	P331962	6/F	Bandenin		12	0	0	168		Neg	Neg	Neg	29.6
	P331983	6/F	Bandenin		36	0	0	5,304		Neg	Neg	Neg	28.1
2017	P341287	61/M	Mendikorma		1,464	0	0	0		Neg	Neg	Neg	33.1
	P341282	56/M	Mendikorma		540	0	216	0		Neg	Neg	28.3	Neg
	P341284	50/M	Mendikorma		60	0	0	132		Neg	Neg	Neg	Neg
	P342148	45/M	Keyabendu		1,368	0	0	192		Neg	Neg	34.5	Neg
	P340246	39/M	Kamatahun		120	0	0	216		Neg	Neg	Neg	30.0
	P340307	19/F	Bambuloe		2,028	0	0	1,188		Neg	Neg	Neg	24.1
	P340133	12/M	Fokolahun		1,020	16,392	0	0		25.3	16.7	Neg	Neg
	P340183	9/F	Kpelloe Ndama		72	0	0	0		Neg	Neg	Neg	36.0
	P341308	9/F	Mendikorma		36	0	108	0		Neg	Neg	Neg	28.5
	P341326	9/M	Mendikorma		456	0	0	0		Neg	Neg	26.5	30.4
	P341327	6/M	Mendikorma		2,076	0	0	0		Neg	Neg	Neg	Neg
	P340147	5/M	Fokolahun		48	0	0	0		30.94	26.93	Neg	Neg

*T. trichiura infection was confirmed by qPCR in only 2 patients, but 25 had *Capillaria* eggs in their stool. Al, *Ascaris lumbricoides*; epg, eggs per gram of stool; Hk, hookworm; Na, *Necator americanus*; Neg, negative; Sm, *Schistosoma mansoni*; Tt, *T. trichiura*.

To check further whether Kato-Katz–positive, qPCR-negative stool samples contained *T. trichiura* eggs, we examined direct smears of stool samples preserved in RNAlater by microscopy (magnification ×100 and ×400) (Figure). The samples positive by qPCR contained eggs (6 measured)with typical *T. trichiura* morphology; these eggs had a mean (±SD) length of 52 µm (±2.4 µm) and width of 25.5 µm (±1.3 µm). In contrast, qPCR-negative samples contained eggs (31 measured) with a mean (±SD) length of 51.8 µm (±1.5 µm) and width of 32.7 µm (±2.1 µm). The qPCR-negative samples also had less pronounced plugs and a thick, striated shell, features that are consistent with eggs of *Capillaria hepatica* (syn. *Calodium hepaticum*) and some other *Capillaria* species (*Trichuridae*). Eggs of *C. philippinensis* or *C. aerophila* that have been observed in human stool samples previously were either smaller or larger than the *Capillaria* eggs found in Lofa (*12*,*13*). Because polar plugs of these eggs are less prominent than those of *T. trichiura*, and because their shapes are sometimes more oval or round, they can also be confused with *A. lumbricoides* eggs by low-power microscopy, especially if only a few eggs were detected (Figure).

**Figure:**
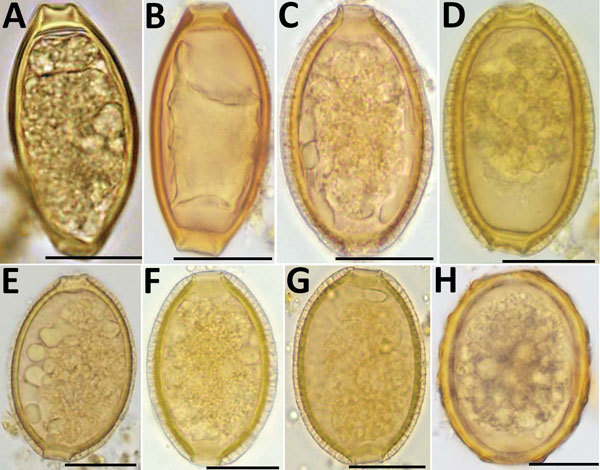
Helminth eggs found in stool samples from persons in Lofa County, Liberia. A, B) Eggs of *Trichuris trichiura* in samples positive for *T. trichiura* by Kato-Katz smear and by qPCR. C–F) Eggs of *Capillaria* spp. in samples positive for *T. trichiura* by Kato-Katz smear but negative for *T. trichiura* by qPCR. G) Egg of *Capillaria* spp. in sample positive for *Ascari lumbricoides* by Kato-Katz smear but negative for *A. lumbricoides* by qPCR. H) Egg of *A. lumbricoides* in sample positive for *A. lumbricoides* by Kato-Katz smear and qPCR. Scale bars indicate 20 µm. qPCR, quantitative PCR.

Members of the subfamily *Capillaridae* are animal parasites with somewhat divergent life cycles, and most do not infect humans. Pseudoinfections with *C. hepatica* occur; eggs found in stool are present because they were consumed in infected animal liver. However, actual infections with *C. hepatica* do not lead to the passing of eggs in stool (*9*). Other species such as *C. philippinensis* cause true infections (and autoinfection) with eggs found in stool; the infection is linked to consumption of raw fish. Human capillariasis has not been reported from Liberia, and only isolated case reports have been published from sub-Saharan Africa (*7*–*9*). We performed DNA sequencing to better characterize the *Capillaria* species found in Foya. Using the primers Kt875351.1 (5′-CCCTAGTTGCGACTTTAAACGA-3′**)** and *Capillaria* 18S1R (5′- TCCACCAACTAAGAACGGCC-3′), we were able to amplify and sequence a 288-bp portion of the 18S rDNA from *T. trichiura* qPCR-negative samples that contained only eggs morphologically identified as *Capillaria* spp. (GenBank accession no. MG859285). The DNA fragment was 100% identical to orthologs of *C. hepatica* (accession no. MF287972.1), *Aonchotheca putorii* (*C. putorii*) (accession no. LC052356.2), and *Pearsonema plica* (*C. plica*) (accession no. MF621034.1), *Capillaria* worm species that have varying life cycles and host species but that are only 95% identical to the ortholog of *T. trichiura*.

The life cycle and the medical importance of the *Capillaria* species found in humans in northwestern Liberia remain to be elucidated. In our study some subjects showed high *Capillaria* egg loads that may indicate a true infection rather than pseudoinfection. However, transient high egg counts have been reported in persons with pseudoinfections (*7*). Whereas consumption of bush meat in Foya is common, consumption of raw or undercooked fish, which is necessary for transmission of *C. philippinesis*, is rare.

## Conclusions

This study shows that *Capillaria* eggs similar to those of *C. hepatica* are not uncommon in stool samples collected in Liberia. These eggs can be misidentified by Kato-Katz smear as *T. trichiura* or as *A. lumbricoides,* which can confound results of STH surveys. The misidentification can also lead to an incorrect assumption that antihelminthic treatment was ineffective. Our results also illustrate the value of qPCR for validating Kato-Katz test results and for explaining unexpected findings.
